# From CMS to iCMS/IMF: Developing Roadmap to Precision Therapy in Colorectal Cancer

**DOI:** 10.3390/ijms262211086

**Published:** 2025-11-16

**Authors:** Sungwon Jung

**Affiliations:** 1 Department of Genome Medicine and Science, Gachon University College of Medicine, Incheon 21565, Republic of Korea; sjung@gachon.ac.kr; Tel.: +82-32-458-2740; 2 Gachon Institute of Genome Medicine and Science, Gachon University Gil Medical Center, Incheon 21565, Republic of Korea

**Keywords:** colorectal cancer, iCMS, IMF, fibrosis, single-cell, CMS

## Abstract

Colorectal cancer (CRC) classification has progressed from consensus molecular subtypes (CMS) to epithelial–intrinsic consensus molecular subtypes (iCMS) and the layered intrinsic subtype-MSI-fibrosis (IMF) system that combines intrinsic state, MSI status, and fibrosis. This article reviews biological underpinnings of iCMS/IMF, their relationships to tumor-microenvironment crosstalk, and how single-cell and spatial transcriptomics refine therapeutic stratification by resolving tumor microenvironment heterogeneity and its impact on fibrosis. Prognostic and therapeutic implications are covered, including PD-1 blockade in MSI-high (MSI-H), MAPK-directed therapy in BRAF-mutant disease, and EGFR targeting in selected RAS wild-type (WT) left-sided tumors, and we suggest decision points specifically informed by the activity of the fibrosis axis. A step-by-step procedure is presented for the analysis of bulk and single-cell RNA and formalin-fixed, paraffin-embedded (FFPE) resources, along with open-source tools and reporting standards to make iCMS/IMF calling reproducible in clinics and trials. Future outlooks are outlined with near-term biomarker–drug hypotheses for microsatellite-stable (MSS)-iCMS3 and high fibrosis tumors and key gaps to close for clinical translation. This review outlines a roadmap for precision medicine in colorectal cancer by leveraging the iCMS/IMF framework to integrate pathology and digital pathology, molecular diagnostics, and therapy mapping with FAP-targeted imaging and therapy.

## 1. Introduction

CRC remains a major cancer mortality and exhibits marked inter- and intra-heterogeneity spanning epithelial mechanisms and the tumor microenvironment (TME). This biological diversity drives wide variability in prognosis and therapeutic response, challenging one-size-fits-all regimens. The CMS framework offered the first coherent transcriptomic taxonomy for CRC, but bulk-tissue confounding by stromal admixture can obscure epithelial–intrinsic signals and limit direct translation. Recent single-cell and spatial transcriptomic studies have delineated reproducible epithelial states and immune–stromal ecosystems that shape treatment opportunities and resistance. From these circumstances, there is a clear need for robust, clinically translatable stratification that cleanly separates epithelial state from MSI status and fibrosis to guide decision-making across disease contexts [1,2,3,4].

The CMS framework unified several earlier expression-based taxonomies into four consensus subtypes with distinct biology and clinical associations, creating a shared language for CRC research and trials [1]. It showed strong portability across platforms and cohorts, yet a nontrivial fraction of tumors (≈13%) remained “mixed or unclassified”, reflecting intratumoral heterogeneity or transitional states [1]. Because CMS relies on bulk RNA from whole-tumor sections, stromal admixture, especially fibroblast-rich mesenchymal programs, can dominate the signal and blur epithelial–intrinsic biology; this is most evident in CMS4, which captures angiogenic, immunosuppressive TME features tied to poor prognosis [5]. To disentangle tumor-cell biology from microenvironmental noise, “intrinsic” approaches such as CRC intrinsic subtypes (CRIS) were derived using human-specific profiling of patient-derived xenografts (PDXs) [6].

In brief, the CRIS taxonomy isolates tumor-cell signals by training on human-specific expression profiles extracted from PDXs [6,7]. CRIS delineates five epithelial programs (CRIS-A to CRIS-E) with distinct biology and potential therapeutic associations: a mucinous/glycolytic program (CRIS-A); a TGF-β/epithelial–mesenchymal transition (EMT)-high mesenchymal program associated with adverse outcome (CRIS-B); an EGFR-activated program that can mark EGFR dependency (CRIS-C); a WNT/IGF2-driven proliferative program (CRIS-D); and a secretory/Paneth-like differentiation program (CRIS-E). Because CRIS explicitly minimizes stromal confounding, it conceptually bridges bulk-defined CMS and the single-cell-derived intrinsic CMS (iCMS) axis; iCMS2/iCMS3 provide a robust binary backbone of epithelial state, while CRIS offers finer granularity within that backbone and helps map actionable epithelial phenotypes (e.g., EGFR-driven CRIS-C) onto the iCMS/IMF framework.

Additionally, orthogonal solutions like image-based CMS (imCMS) predict transcriptomic classes directly from hematoxylin and eosin (H&E) slides, reclassifying previously unassignable or heterogeneous cases and mapping spatial heterogeneity [8]. Together, these observations motivate refinement toward epithelial–intrinsic and layered schemes of iCMS and IMF that explicitly separate epithelial state from MSI status and fibrosis [2] by sequentially assigning the states for these three orthogonal axes.

Building on the limitations of bulk-defined CMS, recent integrative single-cell work delineated a robust epithelial–intrinsic dichotomy, iCMS2 and iCMS3, that forms the tumor-cell backbone of CRC taxonomy. In a compendium of 373,058 single-cell transcriptomes from 63 patients (including 49,155 epithelial cells) and 3614 bulk tumors across 15 cohorts, Joanito et al. [2] showed that iCMS2 is characterized by recurrent arm-level copy-number gains or losses (for example, 7pq, 8q, 13q, 20pq gains), whereas iCMS3 is typically diploid or copy-number light; importantly, iCMS3 encompasses all MSI-H tumors and up to one-third of MSS tumors, with MSS-iCMS3 transcriptomes more similar to MSI-H than to MSS-iCMS2. The analysis also reconciled CMS with “intrinsic” cancer-cell taxonomies such as CRIS, demonstrating that the iCMS axis captures the core epithelial biology obscured by stromal admixture in CMS4 and helps explain the worse prognosis when CMS4 harbors iCMS3 epithelium [2,6]. Complementary single-cell profiling independently linked epithelial lineage programs to immune landscapes in CRC, implying that a tumor-cell-centric classification is biologically grounded and clinically promising [3].

The IMF framework overlays the epithelial–intrinsic backbone (I: iCMS2/iCMS3) with MSI (M) and a quantifiable fibrosis axis (F), yielding five practical subtypes that sharpen both prognostic and predictive resolution beyond bulk-defined CMS; subsequent pathological updates have highlighted its translational appeal [2,9]. By explicitly separating tumor-cell state from MSI status and stromal desmoplasia, IMF explains why F-high tumors, marked by cancer-associated fibroblast (CAF)/TGF-β-driven remodeling and immune exclusion, track with adverse outcomes and therapeutic resistance, nominating microenvironment-modulating strategies as rational combinations [5,10]. This layered biology maps directly to actionable decision points: MSI-H/deficient mismatch repair (dMMR) disease favors PD-1 blockade as a first-line approach [11,12]; BRAF V600E positivity supports encorafenib plus cetuximab in previously treated settings (with first-line expansion now emerging) [13,14]; left-sided RAS-WT tumors derive greater benefit from EGFR-inhibitor-based chemotherapy than from bevacizumab-based regimens [15]; and F-high phenotypes motivate trials of TGF-β pathway inhibition and FAP-targeted theranostics (FAP-inhibitor (FAPI) imaging/therapy) as stroma-focused complements to epithelial-directed therapy [10,16,17].

**Figure 1:**
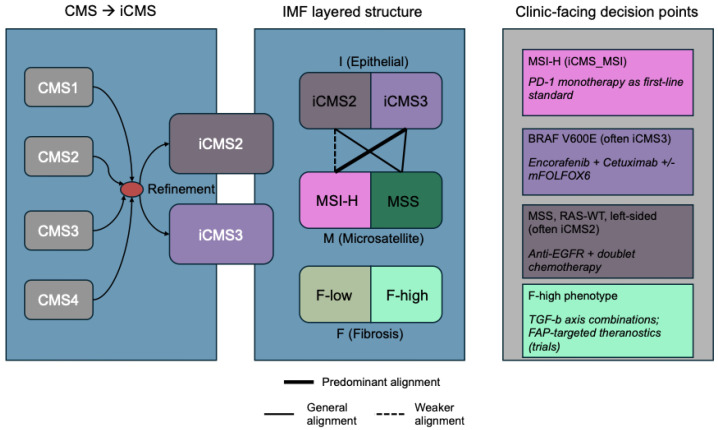
Conceptual overview of the transition from CMS to iCMS/IMF and clinic-facing decision points. The intrinsic epithelial axis (I: iCMS2 vs. iCMS3) is layered with microsatellite status (M: MSI-H vs. MSS) and a fibrosis axis (F: low vs. high). MSI-H predominantly aligns with iCMS3 (solid link), whereas fibrosis (F-high) spans epithelial states and modulates therapeutic sensitivity. The right-hand box summarizes illustrative decision suggestions: PD-1 monotherapy in MSI-H/iCMS3; encorafenib + cetuximab (+/− mFOLFOX6) for BRAF V600E disease; anti-EGFR plus chemotherapy for MSS, RAS-WT, and left-sided tumors; and stroma-modulating strategies (e.g., TGF-β combinations, FAP-targeted theranostics) in F-high phenotypes. Abbreviations: CMS, consensus molecular subtypes; iCMS, epithelial–intrinsic consensus molecular subtypes; IMF, intrinsic subtype-MSI-fibrosis; MSI, microsatellite instability; MSI-H, MSI-high; MSS, microsatellite-stable; mFOLFOX6, modified fluorouracil/leucovorin/oxaliplatin; WT, wild-type.

In this review, we translate the biology of iCMS/IMF into a currently feasible workflow: single-sample calling of iCMS2/iCMS3 from bulk tumor RNA, integration of routine MSI testing, and quantitative fibroblast/fibrosis readouts (e.g., estimate the proportion of immune and cancer cells (EPIC) [18], microenvironment cell populations (MCP)-counter [19]) with transparent quality control (QC) and reporting checklists [2,5,18]. To broaden real-world applicability, we include FFPE-optimized and image-based surrogates (FFPE-curated CMS classifier (CMSFFPE) [20], imCMS) so centers without fresh-frozen RNA-seq can still run subtype-informed procedures [8,20]. Finally, we map IMF groups to actionability, PD-1 blockade for MSI-H/dMMR disease, encorafenib–cetuximab for BRAF V600E tumors, and microenvironment-modulating opportunities (e.g., TGF-β targeting) for F-high phenotypes, illustrated by a conceptual diagram (Figure 1) and resources for trial design and tumor boards [10,11,13,15,16].

## 2. Concepts and Definitions: From CMS to iCMS to IMF

The CMS unified earlier expression-based schemes into four reproducible classes with distinct biology and clinical associations, CMS1 (MSI-immune), CMS2 (canonical/WNT-MYC), CMS3 (metabolic/KRAS-enriched), and CMS4 (mesenchymal/TGF-β/angiogenic), and quickly became a popular concept for CRC research and trial design [1]. Its strengths include cross-platform portability and a coherent link between pathway activation and outcomes. Yet CMS classifications are derived from bulk tumor transcriptomes, where stromal admixture—particularly the fibroblast-rich, TGF-beta-driven biology characteristics of CMS4—can dominate the signal. This often masks epithelial-intrinsic features and leads to “mixed or unclassifiable” labels, complicating direct clinical decision-making [1,5]. Efforts to isolate cancer-cell signals (e.g., the CRIS intrinsic taxonomy) and to infer CMS from images (imCMS) illustrate two complementary responses to these bulk limitations: one strips away stromal noise to reveal tumor-cell biology, while the other maps spatial heterogeneity that bulk averages obscure [6,8].

Leveraging large single-cell atlases integrated with matched bulk cohorts, Joanito et al. [2] delineated a robust epithelial dichotomy of iCMS2 and iCMS3, where it recapitulates in bulk tumors and cleanly separates tumor-cell biology from microenvironmental noise. iCMS states are distinguished by transcriptional programs, copy-number architectures (iCMS2 tending toward arm-level aneuploidy; iCMS3 often diploid or copy-number-light), and regulatory-network features. More importantly, MSI-H tumors cluster almost entirely within iCMS3, while a biologically coherent subset of MSS-iCMS3 resembles MSI-H more than MSS-iCMS2 [2]. Independent single-cell work further links epithelial lineage biology to immune landscapes in CRC, reinforcing the biological grounding of an epithelial–intrinsic backbone for classification and hypothesis generation [3].

To convert epithelial–intrinsic biology into a clinic-facing taxonomy, the IMF framework overlays the intrinsic epithelial state (I: iCMS2 vs. iCMS3) with microsatellite status (M) and a quantifiable fibrosis axis (F). This yields five practical groups (for example, iCMS2_MSS_NF, iCMS2_MSS_F, iCMS3_MSS_NF, iCMS3_MSS_F, and iCMS3_MSI) that reconcile bulk CMS labels with tumor-cell biology and stromal context [2]. Notably, fibrosis is orthogonal to the epithelial backbone: The fibrotic CMS4 compartment contains both iCMS2 and iCMS3 epithelium, yet the worst outcomes concentrate in fibrotic tumors with iCMS3 epithelium, a finding corroborated by multiregional profiling that refines prognostication within iCMS3 [2,21]. Mechanistically, iCMS3_MSS_F exhibits enriched fibroblast, endothelial, and myeloid biology, as well as immune exclusion and desmoplastic features consistent with metastatic propensity and inferior relapse-free survival [2]. Figure 1 illustrates this transition from CMS to iCMS/IMF and layers I (iCMS2/iCMS3), M (MSI-H/MSS), and F (low/high) into a single clinic-facing view.

The CRIS taxonomy, derived by extracting human cancer-cell expression from PDXs, defined five epithelial programs with prognostic and predictive relevance—an early move to decouple tumor-cell signals from stroma [6]. iCMS, in contrast, emerges from large-scale single-cell integration and condenses tumor-cell diversity into a robust two-state backbone (iCMS2 and iCMS3) that can be layered with MSI and fibrosis in IMF [2]. The schemes are complementary: iCMS provides a stable binary axis, whereas CRIS supplies finer-grained epithelial phenotypes, where their comparisons show concordance but not a 1-to-1 mapping, reflecting true biological variance. For translation when RNA-seq is impractical, imCMS infers transcriptomic classes directly from H&E and exposes spatial heterogeneity [8], and CMSFFPE enables FFPE-optimized bulk classification with documented clinical portability [20]. Together with multiregional analyses that quantify subtype heterogeneity in situ [21], these tools position iCMS/IMF as the key concept regarding epithelium, CRIS as refinement within the key concept, and imCMS/CMSFFPE as pathology-ready surrogates for real-world deployment.

## 3. The Fibrosis Axis and the Contributions of Single-Cell and Spatial Transcriptomics

CRC harbors diverse CAF states, including IL1R1^+^, IL-1-high CAFs that suppress T cells and macrophages, converging on extracellular matrix (ECM) remodeling, stromal stiffening, and immune exclusion [22]. Central to this ecology, TGF-β and SMAD signaling from fibroblasts, myeloid cells, and tumor epithelium drives ECM deposition, angiogenesis, and cytotoxic T-cell exclusion, while in colon cancer models, TGF-β blockade converts “cold”, excluded tumors into checkpoint-responsive lesions [23,24]. Clinically, this underpins combination strategies pairing immunotherapy with TGF-β pathway inhibitors [10] and enables stromal readouts via FAP-targeted PET (FAPI-PET), which highlights fibroblast-rich compartments (e.g., CMS4-like and peritoneal metastases) and is being explored for theranostics [16,25].

Spatial transcriptomics shows that different neighborhoods within a single tumor can map to different CMS classes, with CAF- and immune-rich regions driving CMS4-like signals adjacent to epithelial islands that align with CMS2/3, clarifying when bulk labels reflect stromal admixture rather than uniform epithelial state [26]. Complementary multiregional transcriptomics quantifies subtype mixing and introduces congruent CMS assignments that better explain survival variation than heterogeneity alone, supporting region-aware subtyping for prognosis and trial design [21]. Integrative spatial transcriptomics with single-cell analyses further recover ligand–receptor circuits at the tumor–stroma interface, linking spatial architecture to intercellular communication and therapeutic hypotheses in CRC [27].

Single-cell and spatial atlases reveal multicellular “immune hubs” that organize antitumor activity and associate with immunotherapy sensitivity: a dMMR-enriched intratumoral hub with activated T cells and chemokine-expressing malignant/myeloid cells, and a myeloid-attracting hub at the luminal edge tied to tissue damage [4]. Extending this concept, the tumor–stroma boundary emerges as a decisive niche. In immune-checkpoint blockade responders, LAMP3^+^ dendritic cells engage CXCL13^+^ T cells across a contiguous boundary, whereas non-responders show CXCL14^+^ CAF barriers and discontinuous infiltration where boundary organization distinguishes dMMR (MSI-H) from proficient mismatch repair (pMMR) CRC and tracks with immune-checkpoint blockade outcomes [28]. Spatiotemporal single-cell profiling corroborates response-associated cellular dynamics, nominating boundary-targeted strategies such as DC recruitment and TLS induction as well as CAF-TGF-β remodeling to convert non-responders [29].

## 4. Therapeutic Mapping: Decision Points by iCMS and IMF

**Figure 2:**
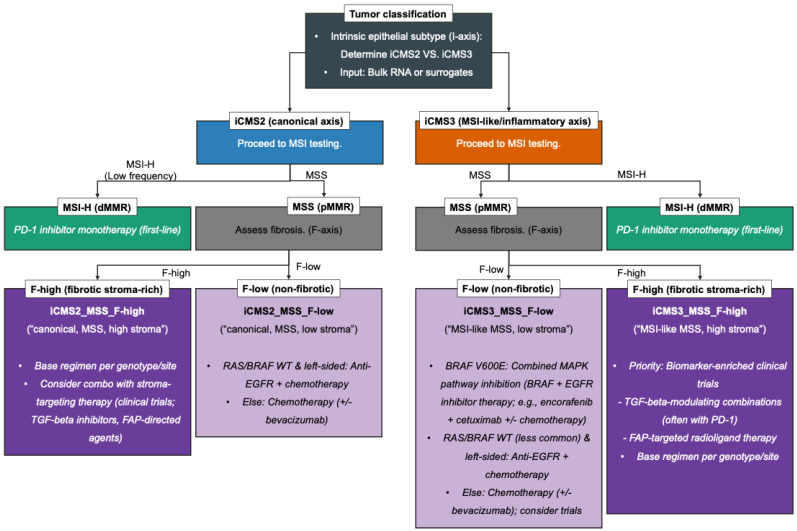
Therapeutic roadmap integrating iCMS (I), MSI (M), and fibrosis (F) for colorectal cancer. The flowchart sequentially stratifies tumors by the intrinsic epithelial axis (iCMS2 vs. iCMS3), MSI status (MSI-H vs. MSS), and fibrosis (F-high vs. F-low), linking each path to actionable treatments. MSI-H disease is directed to first-line PD-1 blockade. In MSS, genotype and sidedness inform targeted options (e.g., encorafenib + cetuximab +/− mFOLFOX6 for BRAF V600E; anti-EGFR + chemotherapy for RAS-WT, left-sided tumors). F-high phenotypes highlight stroma-modulating strategies (e.g., TGF-β pathway inhibition, FAP-targeted theranostics) frequently in the context of clinical trials. Abbreviations: iCMS, epthelial-intrinsic consensus molecular subtypes; MSI, microsatellite instability; MSI-H, MSI-high; MSS, microsatellite-stable; dMMR, deficient mismatch repair; pMMR, proficient mismatch repair; WT, wild-type; mFOLFOX6, modified fluorouracil/leucovorin/oxaliplatin.

The right-hand panel of Figure 1 summarizes the illustrative decision points referenced throughout Section 4.1, Section 4.2, Section 4.3, Section 4.4 and Section 4.5, and Figure 2 presents a stepwise therapeutic decision flow integrating the iCMS/IMF framework—linking iCMS, MSI status, and fibrosis to specific, actionable treatments and trial priorities. An evidence summary is listed in Table 1.

### 4.1. MSI-H/iCMS3: First-Line PD-1 Blockade and Resistance Considerations

Among IMF groups, MSI-H disease, largely on the iCMS3 epithelial state, has a first-line SOC with PD-1 blockade. In the KEYNOTE-177 study, first-line pembrolizumab delivered superior progression-free survival versus chemotherapy (median OS 77.5 vs. 36.7 mo (HR 0.73), PFS 16.5 vs. 8.2 mo (HR 0.60)) with fewer grade ≥ 3 AEs, establishing PD-1 monotherapy as the preferred initial strategy in MSI-H/dMMR mCRC [11,12]. With extended follow-up, OS and duration-of-response advantages persisted despite substantial crossover to immunotherapy, reinforcing durable benefit [30]. Mechanistically, MSI-H aligns with iCMS3, helping explain the strong immunotherapy signal in this subgroup [2]. Still, primary and acquired resistance occur. Reviews highlight antigen-presentation defects, interferon-γ-pathway lesions, microenvironmental exclusion, and microbiome factors while noting that B2M or JAK1/2 loss-of-function does not uniformly predict non-response in CRC, unlike in some other cancers [31,32]. For the practice note, recent studies and reports suggest PD-1 monotherapy for MSI-H/iCMS3, and at progression, clinical trials and rational PD-1-based combinations can be favored, recognizing that randomized first-line evidence supports pembrolizumab monotherapy [2,11,12,30,31,32].

### 4.2. iCMS3_MSS: MSI-H-like Biology and Combination-Immunotherapy Hypotheses (Trial Priorities, Not Standards)

Within the iCMS scaffold, a subset of MSS tumors with iCMS3 epithelium transcriptionally resembles MSI-H disease, suggesting that immune-focused strategies may succeed if the microenvironment can be favorably remodeled [2]. Early clinical signals are mixed: The VEGF-multikinase/PD-1 doublet regorafenib + nivolumab achieved a modest response rate in a multicenter phase II and appeared context-dependent (responses concentrated in patients without baseline liver metastases) [33]. By contrast, randomized trials at immunologic “heating” of MSS CRC (atezolizumab + cobimetinib (IMblaze370) [34]; lenvatinib + pembrolizumab (LEAP-017) [35]) did not improve survival over standard options. Beyond ICIs, T-cell-redirecting approaches such as the CEA × CD3 bispecific cibisatamab showed proof-of-mechanism but with immunogenicity and gastrointestinal on-target toxicity that mandate careful selection and combinations (e.g., B-cell depletion) [36]. Biomarker hypotheses to enrich iCMS3_MSS for trials include CEACAM5-high expression for T-cell engagers, absence of liver metastases for VEGF/PD-1 regimens, and rare hypermutated MSS driven by POLE/POLD1 or very high tumor mutational burden (TMB) (exceptions that can respond to ICIs) [33,37,38]. These currently available reports suggest that trial enrollment, rather than off-protocol use, remains an approachable option for iCMS_MSS.

### 4.3. MAPK/BRAF V600E (Often iCMS3): Targeted Therapy and First-Line Expansion

BRAF-V600E-mutant mCRC frequently occurs within the iCMS epithelial state, a context aligned with a CpG-island methylator phenotype (CIMP-high), BRAF-associated molecular phenotype, and characteristic immune–stromal biology [2,9]. In CRC, BRAF inhibition alone is inadequate because EGFR-mediated feedback restores MAPK signaling; effective strategies therefore co-inhibit BRAF and EGFR (±MEK) [39]. The phase-III BEACON program established encorafenib + cetuximab (EC), with or without binimetinib, as superior to irinotecan-based controls after prior therapy, defining the EC doublet as standard in the pretreated setting [13,14]. Additionally, the phase-III BREAKWATER trial showed that EC + mFOLFOX6 significantly improved PFS (12.8 vs. 7.1 mo (HR 0.53)) and OS (30.3 months vs. 15.1 months (HR 0.49)) versus standard chemotherapy, with safety consistent with known profiles [40]. On the strength of interim efficacy, the U.S. Food and Drug Administration (FDA) granted accelerated approval to EC + mFOLFOX6 for BRAF-V600E mCRC, including first-line use pending confirmation, which BREAKWATER’s survival data now support [40,41]. These can be summarized as the following practice note: for pMMR/MSS BRAF-V600E mCRC, EC + mFOLFOX6 can be a first-line targeted option supported by phase-III survival benefit, and for MSI-H, PD-1 blockade may remain the default initial strategy, with BRAF-targeted therapy considered at progression.

### 4.4. iCMS2 (Canonical Axis): The EGFR-First Principle in RAS-WT, Left-Sided Disease

Patients who are MSS, RAS-WT, and left-sided, a group that often aligns with canonical epithelial states (CMS2/iCMS2), derive first-line benefit from adding an anti-EGFR antibody to doublet chemotherapy. The phase-III PARADIGM trial (mFOLFOX6 + panitumumab vs. mFOLFOX6 + bevacizumab) showed OS superiority in left-sided RAS-WT mCRC, with higher response and greater curative-resection rates, establishing an EGFR-targeting practice when conversion, symptom control, and long-term survival are priorities [42]. A meta-analysis of head-to-head first-line trials confirmed this pattern in left-sided RAS-WT disease (OS advantage and higher ORR) and highlighted the absence of OS benefit on the right side, where PFS often favors bevacizumab-based therapy [15]. Guidelines and expert reviews position doublet chemotherapy + anti-EGFR as the preferred first-line option for MSS, RAS-WT, and left-sided mCRC, refined by BRAF status and IMF layers (e.g., MSI-H handled per the direction in Section 4.1, and F-high biology per the direction in Section 4.5) [43,44].

### 4.5. F-High Phenotypes: Anti-Fibrosis/TGF-β Strategies and FAP Theranostics

Within IMF, F-high (desmoplastic) phenotypes, notably iCMS_MSS_F, are enriched for CAF/TGF-β-driven mechanisms that stiffen ECM, exclude cytotoxic T cells, and foster drug resistance, making stroma-modulating therapy a rational co-strategy alongside epithelial- or immune-directed treatments [10,24]. On the TGF-β axis, multiple therapeutic classes are in development, including neutralizing antibodies, ALK5 kinase inhibitors (e.g., vactosertib and galunisertib), and ligand traps/bifunctionals, with the mechanistic goal of decompressing stroma and reversing immune exclusion, and CRC-focused reviews emphasize context-dependent biology and the need for combinations rather than monotherapy [10,24]. In parallel, FAPI-PET has emerged as a sensitive, low-background imaging readout of stromal activation, improving detection of peritoneal and liver metastases and enabling patient selection/monitoring in stroma-high CRC [16,45]. Important caveats include benign fibro–inflammatory uptake and short tumor retention for first-generation tracers, motivating next-generation ligands and careful interpretation [45,46,47]. On therapy, FAP-targeted radioligand therapy (TRT) has shown preliminary antitumor activity with manageable safety in early studies, while ongoing programs (e.g., LuMIERE) and higher-retention ligands (e.g., OncoFAP-23) address residence time, dose delivery, and organ safety [17,48,49]. Representative next-generation FAP ligands and ongoing FAP-TRT programs are summarized in Table 2. These reports and studies suggest the following practice approach: in iCMS3_MSS_F or other F-high IMF groups, considering trial enrollment for TGF-β-modulating combinations (often with PD-1) and FAPI-guided theranostics, using FAPI-PET chiefly as a biomarker or eligibility tool until randomized data mature.

## 5. Suggested Implementation Guide: Calling iCMS/IMF in Clinical and Research Cohorts

### 5.1. Quality Control Prerequisites for IMF Classification Pipeline

Before applying any IMF subtyping, both bulk and single-cell RNA-seq data must pass rigorous QC. For bulk RNA-seq, start with high-quality RNA (RNA integrity number (RIN) ≥ 7 to ensure intact RNA) and verify sufficient tumor content in the sample. After sequencing, evaluate read quality and alignment: At least 70–80% of reads should map uniquely to the human reference genome (mapping rates much below 70% often indicate poor sample quality or contamination). Check library complexity by measuring duplicate reads—excessive PCR duplication skews expression data, so the duplication rate should be kept low. It is also important to confirm even coverage across transcripts (e.g., uniform gene body coverage with minimal 5′ or 3′ bias). Standard QC tools such as FastQC [50], Picard [51], and RSeQC [52] can generate these metrics. Only when a bulk RNA-seq library meets these QC benchmarks (high RIN, robust mapping rate, low duplicates, broad gene coverage) should it proceed to normalization and iCMS classification.

For single-cell RNA-seq, QC is performed at the cell level to filter out low-quality or artifactual cells. Typical criteria remove cells with extremely small or large transcript counts—for example, discard barcodes with fewer than ~200 detected genes (likely empty droplets) and consider an upper unique molecular identifier (UMI)/gene cutoff to catch potential doublets. Cells with abnormally high mitochondrial signals may be stressed or dying. In addition, specialized algorithms are applied to identify multicell capture events: Tools like Scrublet [53] and DoubletFinder [54] flag probable doublets so they can be excluded from analysis. These cell-level QC steps (gene count filters, mitochondrial content cutoff, doublet removal) are essential to obtain reliable single-cell expression data for downstream analysis. Finally, to ensure reproducibility, all QC thresholds and software tools used (with versions and parameters) should be clearly documented in methods or reports. This transparency in reporting enables consistent IMF analysis across studies and clinical applications.

### 5.2. Inputs and Step-by-Step Pipeline to Call IMF (I + M + F)

**Figure 3:**
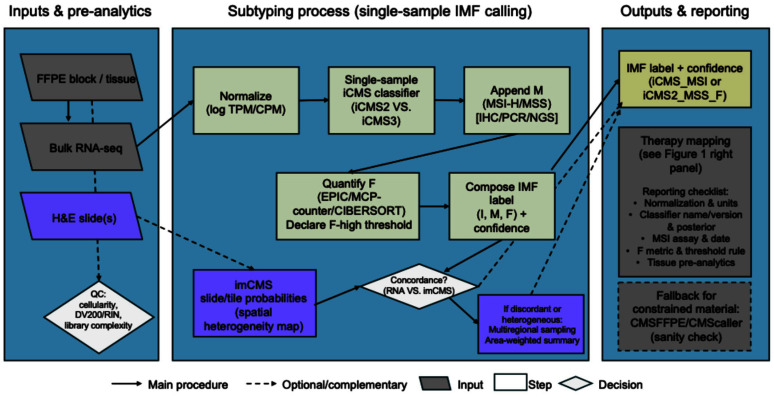
Suggested pipeline to call iCMS/IMF from routine materials. RNA-seq data is normalized and processed with a single-sample iCMS classifier (I), layered with MSI testing (M), and fibroblast/fibrosis quantification (F; EPIC/MCP-counter/CIBERSORT [56] with prespecified F-high threshold) to compose an IMF label with confidence. In parallel, H&E-based imCMS provides slide/tile-level probabilities and spatial heterogeneity; concordance checks trigger multiregional sampling and area-weighted summaries when needed. Outputs include an IMF label and a reporting checklist to ensure reproducibility; therapy mapping is summarized in Figure 1 (right-hand panel). Abbreviations: iCMS, epithelial–intrinsic consensus molecular subtypes; IMF, intrinsic subtype-MSI-fibrosis; FFPE, formalin-fixed, paraffin-embedded; H&E, hematoxylin and eosin; QC, quality control; RIN, RNA integrity number; TPM, transcript per million; CPM, counts per million mapped reads; MSI, microsatellite instability; MSI-H, MSI-high; MSS, microsatellite-stable; IHC, immunohistochemistry; PCR, polymerase chain reaction; NGS, next-generation sequencing; EPIC, estimate the proportion of immune and cancer cells; MCP, microenvironment cell populations; imCMS, image-based CMS.

The end-to-end workflow is summarized in Figure 3. Normalize expression (e.g., log-applied TPM or CPM), run a single-sample iCMS classifier to determine the intrinsic epithelial state (I: iCMS2 vs. iCMS3), and report it along with the classifier name/version, input units, and assignment confidence (posterior score or distance-to-decision boundary) for necessary process management [2,55]. Next, append the microsatellite layer (M) using routine clinical testing, immunohistochemistry (IHC) for MMR proteins, polymerase chain reaction (PCR)-based MSI, or next-generation sequencing (NGS) panels, and record the assay, thresholds, and testing date (to manage temporal block discordance). Finally, quantify the fibrosis/CAF axis (F) from the bulk profile via deconvolution, e.g., EPIC fibroblast proportion or MCP-counter fibroblast score, and pre-declare a rule for F-high vs. F-low (cohort median, external cut-point, or percentile-based threshold) with sensitivity analyses for robustness [5,18]. The output is an IMF composite label (e.g., iCMS3_MSI or iCMS3_MSS_F), plus a brief interpretive note (biological hallmarks and likely “decision points”) and a methods box listing the following: data source, normalization, classifier/version, MSI method, F metric/threshold, and links to used code/resources [2,5,18,55].

All classification tools used are openly available and documented. For instance, the single-sample iCMS classifier from Joanito et al. is provided as an R package iCMS.SSC version 0.9 on GitHub [55] (installable via devtools, as no formal CRAN/Bioconductor release exists). This iCMS package is actively maintained, with recent code commits in 2024–2025 ensuring compatibility. Likewise, legacy CMS subtyping can be performed with the open-source CMScaller tool—an R package (GPL-3.0 license) available on GitHub [57] that implements a nearest template prediction algorithm for CMS assignment. While CMScaller repository has not seen updates beyond mid-2020, it includes a comprehensive user guide and vignettes and remains fully accessible for reproducible CMS classification.

### 5.3. FFPE/Low-Quality Material: Compatibility and Specialized Classifiers

When only FFPE or otherwise degraded/low-input RNA is available, it is recommended to maintain subtype fidelity with classifiers tailored to fixation-induced fragmentation and 3′ bias. An FFPE-optimized workflow, CMSFFPE, uses a curated gene set and training on FFPE-like profiles to deliver CMS calls with high concordance to fresh-frozen RNA-seq, and it functions best as a supportive readout alongside iCMS/IMF when bulk RNA-seq permits intrinsic-state calling [20]. In practice, it is recommended to report pre-analytic variables (block age, fixation, DV200), library type (e.g., 3′ counting or targeted capture), and the classifier/version with call confidence. For legacy microarrays, targeted panels, or shallow RNA-seq, single-sample CMS tools such as CMScaller [58] are valuable for sanity checks and for preclinical/archival sets, while recognizing they recapitulate CMS (not iCMS) and should not be treated as a one-to-one surrogate for epithelial-state labels. It is recommended to keep outputs actionable by co-reporting FFPE-derived CMS with any available iCMS/IMF call, adding a short concordance note when labels disagree, and documenting any thresholds/batch handling applied.

In practice, pre-analytical variables such as tumor cellularity, block age, and tumor area can critically impact the success of using FFPE-based classifications. Pathology recommendations advise enriching samples to ensure a sufficient tumor fraction—generally at least 20–30% tumor cell content in the analyzed region—for reliable RNA expression profiling [59]. Likewise, the archival age of FFPE blocks should be limited, as RNA integrity degrades over time; notably, studies have observed markedly diminished RNA signals in blocks older than roughly 5 years [60]. Regarding H&E slide for image analysis (which will be mentioned in Section 5.4), a minimum tumor area is required to capture representative morphology. In practice, having on the order of tens of square millimeters of viable tumor (approximately one low-power field, ~25 mm^2^) is recommended to yield a robust classification. Adhering to these pre-analytical criteria—adequate tumor content, acceptable block age, and sufficient tumor area—will help maximize the accuracy and reproducibility of molecular and image-based subtyping from FFPE specimens.

### 5.4. Complementary Modalities: Digital Pathology and Multiregional Profiling

When whole-tumor RNA-seq is infeasible or heterogeneity is suspected, it can be better to pair digital pathology with region-aware sampling. imCMS infers CMS directly from H&E slides using deep learning, producing tile-level probability maps that help to utilize mixed or unclassifiable bulk-RNA cases, expose spatial heterogeneity (e.g., CMS4-like stromal neighborhoods adjacent to CMS2/3-like epithelial islands), and help triage macrodissection or sequencing when tissue is limited. Nevertheless, it is better to treat imCMS as a complementary surrogate, not a replacement for iCMS/IMF, by co-displaying slide-level confidence and the fraction of CMS4-like area, then utilize its result with RNA-based calls [8]. If classifications from bulk RNA-seq and imCMS disagree, if fibrosis-associated features (F-high) are spatially heterogeneous, or if primary and metastatic sites yield different labels, perform multiregional transcriptomic profiling, sampling at least three spatially distinct regions (e.g., one central core and two peripheral sectors). Region-wide calling enables an area-weighted summary and a “congruent CMS” measure that carries prognostic value beyond heterogeneity alone. And it can be helpful to add a brief reliability statement when discordance persists, and to make the final IMF assignment to the best-quality RNA sample while preserving spatial context for tumor boards [21].

Besides digital pathology and multiregional sampling, single-cell RNA sequencing can further support IMF calls as a research-level validation tool. While the single-sample iCMS classifier cannot be applied directly to single-cell data (being trained on bulk profiles), single-cell analysis provides orthogonal confirmation of each IMF component. For instance, in a tumor labeled F-high by bulk RNA, the single-cell data should reveal abundant cancer-associated fibroblasts—identifiable by markers like FAP, ACTA2, PDGFRB, and PDPN—confirming a desmoplastic stroma [61]. Likewise, the tumor’s epithelial cells can be examined to see if they predominantly exhibit an iCMS2 vs. iCMS3 expression program, as expected from the bulk subtype call, or if both states co-exist (indicating heterogeneity). Although not needed for routine classification, such single-cell profiling validates the bulk-derived label: It checks that an iCMS3 tumor’s cells truly have iCMS3-like features and that an F-high tumor truly contains a high fraction of CAFs. Moreover, if integrated with spatial data, single-cell results can map fibroblast-rich regions and immune-exclusion zones, explaining how a high-F fibrosis phenotype manifests in situ. In essence, single-cell and spatial analyses are complementary—they reinforce the bulk IMF assignment by zooming into the cellular level, even though the IMF framework itself is defined and executed on bulk tissue.

## 6. Subtype Concordance Across Sites, Organ-Specific Context, and Patient-Derived Organoid-Stroma Translation

In practice, organ- and region-level sampling should quantify how stable a tumor’s subtype signal is across space, not merely whether heterogeneity exists. For each primary/metastatic site, per-region subtype calls (RNA-based and/or imCMS from H&E) can be assigned, and then they can be summarized with an area-weighted profile and a single congruent-subtype measure that captures the dominant signal. Multiregional analyses show that such “congruent CMS” summaries outperform raw heterogeneity counts in prognostic separation, indicating that the persistence of a subtype across regions, rather than the mere presence of mixing, carries the strongest clinical information for survival and trial stratification [21]. Spatial transcriptomics further clarifies that bulk mesenchymal calls often reflect CAF- and immune-enriched neighborhoods adjacent to epithelial islands of other classes, explaining site-to-site and block-to-block variability [26]. imCMS complements this workflow by mapping subtype probabilities directly on slides [8].

The anatomic site of disease modulates immune–stromal ecology and can shape therapeutic readouts. In pMMR/MSS mCRC, the VEGF-multikinase/PD-1 doublet regorafenib + nivolumab showed modest activity overall, but responses clustered in patients without baseline liver metastases, illustrating how a liver-specific microenvironment may blunt immune efficacy and why site stratification matters in cohort design [33]. For stroma-rich peritoneal disease, FAPI-PET provides an F-axis imaging readout with high tumor uptake and low background, improving visualization of small or flat implants and supporting case selection and response monitoring when conventional imaging is equivocal [16,45]. Pitfalls include benign fibro–inflammatory uptake and short tumor retention with first-generation tracers, motivating cautious interpretation and, where available, next-generation ligands [47,62]. These suggest a practical implication of prespecifying analyses by metastatic site (e.g., liver vs. peritoneum), incorporating FAPI-PET as an optional stromal biomarker, and reporting organ-specific efficacy to avoid diluting context-dependent signals.

In addition, patient-derived organoids (PDOs) provide a tractable, patient-matched platform to test therapies under conditions that preserve tumor-intrinsic programs. When co-cultured with matched stromal cells (PDO-stroma), they also retain microenvironmental cues relevant to the F-axis. A large organoid-stroma biobank showed that epithelial states aligned with CRC subtyping and that adding fibroblasts uncovered subtype-contingent drug sensitivities, enabling individualized response assessment in a setting that better mirrors desmoplastic biology [63]. Independent translational studies demonstrated that PDOs can model real-world treatment responses and predict chemotherapy sensitivity in mCRC, supporting their use as a functional bridge from taxonomy to therapy selection and trial prioritization [64,65]. Considering these recent advancements, we can consider documenting genomic concordance between PDO and tumor, reporting drug-response endpoints with reproducibility metrics (area under curve (AUC) or growth rate (GR), and technical replicates), specifying stroma composition (CAF source, ratio, media), and stating turnaround time relative to clinical decision windows, while limitations (limited immune components, site-specific biases, and inter-lab variability) should be acknowledged.

## 7. Discussion

This review integrates recent single-cell and spatial insights to argue that iCMS provides the principal epithelial axis for CRC classification and that IMF, which layers intrinsic epithelial state with microsatellite status and a quantifiable fibrosis axis, offers a clinic-facing framework for prognosis and therapeutic decision-making. Building on CMS as a cross-cohort framework [1], iCMS2 and iCMS3 separate tumor-cell mechanisms from microenvironmental admixture [2], while IMF links those intrinsic states to MSI and stromal desmoplasia in a way that aligns with observed outcome differences and treatment sensitivities. These concepts are then translated into a pipeline for single-sample subtyping from bulk RNA (with FFPE-compatible and image-based surrogates), IMF groups are mapped to established and emerging therapies, and a potential reporting framework is suggested to improve reproducibility from discovery cohorts to tumor boards.

The conceptual shift from CMS to iCMS/IMF is complementary to earlier “intrinsic” approaches and pragmatic surrogates. CRIS demonstrated that cancer-cell programs can be resolved by minimizing stromal signal using PDX-informed training [10], whereas iCMS distills tumor-cell diversity into a robust two-state axis derived directly from single-cell and bulk compendia [2]. When whole-tumor RNA-seq is infeasible, imCMS (deep learning on H&E) and CMSFFPE (FFPE-tuned gene set) provide operational workarounds for case triage and provide the benefit of reconciling mixed or unclassifiable labels [11,30]. At the same time, multiregional and spatial profiling show that CMS4-like signals can arise from CAF- and immune-enriched neighborhoods adjacent to other epithelial states, explaining site-to-site variability and reinforcing why an epithelial-first axis layered with MSI and fibrosis is the right abstraction for clinical translation [21,26].

Beyond tissue-based multiregional and spatial strategies, emerging liquid biopsy approaches—particularly circulating tumor DNA (ctDNA)—offer noninvasive, real-time windows into spatial and temporal heterogeneity in cancer. Unlike a single-region tissue biopsy that provides only a static snapshot, ctDNA fragments in the bloodstream originate from multiple tumor sites (primary and metastatic) and turn over within hours—thus offering a composite, real-time view of spatial genomic heterogeneity that tissue sampling may miss [66,67]. Beyond conventional mutation profiling, new ctDNA-based techniques leverage cell-free DNA (cfDNA) “fragmentomics” and epigenomic signatures to further characterize tumors: For example, analysis of cfDNA fragment size patterns and nucleosome footprints can reveal tumor-specific signals even at a low tumor burden [68], while DNA methylation assays have achieved high accuracy in classifying tumors by tissue of origin [69]. Other circulating analytes—including circulating tumor cells, exosome-derived nucleic acids, and various cell-free RNAs—are also under exploration to capture additional facets of tumor heterogeneity [67]. These liquid biopsy strategies offer key advantages (minimal invasiveness, repeatability for longitudinal tracking, and multi-site tumor representation), but important limitations persist: ctDNA often constitutes only a minor fraction of total cfDNA (especially in early-stage disease), limiting sensitivity, and liquid biopsies lack the spatial resolution and histopathological context of tissue examination [66,70]. Nonetheless, continued advances are rapidly bringing these approaches into clinical translation, where they are expected to complement tissue-based profiling and enhance real-time, multi-focal management of heterogeneous tumors.

Recent studies have demonstrated that the IMF classification provides a more clinically useful stratification of colorectal cancers than intrinsic subtyping (iCMS) with MSI status alone [2]. The IMF system combines three independent tumor features into a layered model. This three-tier scheme stratifies CRCs into five distinct subtypes (iCMS2_MSS_NF, iCMS2_MSS_F, iCMS3_MSS_NF, iCMS3_MSS_F, and iCMS3_MSI). By integrating the fibrosis axis, IMF directly addresses a key shortcoming of earlier two-factor models (iCMS + MSI)—namely, the omission of the tumor microenvironment’s impact on clinical outcome.

Biologically, the IMF system solves the problem of heterogeneity within the iCMS groups that MSI status alone could not capture. Fibrosis (a surrogate for desmoplastic stromal infiltration, as seen in CMS4 tumors) was found to be orthogonal to the intrinsic epithelial subtype [2]. In practice this means that both “stem-like” iCMS3 and “differentiated” iCMS2 tumors can independently develop a high-fibrosis (CMS4-like) stroma. It was identified that only the fibrosis-rich iCMS3 subset had markedly worse prognosis, whereas iCMS classification by itself was not prognostic [2,21]. In fact, the binary iCMS scheme (iCMS2 vs. iCMS3) alone failed to distinguish high-risk cases because iCMS3 encompassed both aggressive and more indolent tumors [21]. The addition of the F-axis uncovered a high-risk subset—fibrotic iCMS3 MSS tumors—which comprised ~14% of all CRCs and drove the worse relapse rates. In contrast, iCMS3 tumors lacking fibrosis showed more moderate outcomes, often with active immune signatures, emphasizing that stromal context is a critical modifier of the tumor’s behavior.

Statistical evidence emphasizes the added potential prognostic value of incorporating the fibrosis dimension. Fibrotic CMS4-classified tumors overall had significantly inferior relapse-free survival in large cohorts (HR = 1.8 vs. non-fibrotic, *p* = 10 × 10^−10^) [2]. Crucially, within the fibrotic category, iCMS3 tumors fared significantly worse than fibrotic iCMS2 tumors (HR = 1.68, *p* = 0.004). Patients with iCMS3_MSS_F tumors (intrinsically de-differentiated epithelium plus high fibrosis) had over twice the risk of relapse compared to all other subtypes (HR = 2.08, *p* = 3.8 × 10^−9^). By contrast, the intrinsic subtypes with no or low fibrosis did not show such dramatic differences in outcomes, explaining why iCMS + MSI alone lacked prognostic power. These findings validate that the IMF classification more sharply defines prognostic groups than either iCMS or iCMS + MSI status alone. In essence, the fibrosis axis captures an aggressive tumor microenvironment phenotype (high cancer-associated fibroblast and stromal content) that confers additional risk beyond genetic subtype and MSI status.

Clinically, the superior stratification achieved by IMF carries direct therapeutic implications. This integrated system delineates biologically distinct tumor subgroups that align with differential therapy responses. Notably, the IMF schema cleanly isolates the MSI-high, immune-enriched tumors (iCMS_MSI), which correspond to the CMS1 phenotype characterized by abundant cytotoxic lymphocytes [5]. This subgroup is known to derive benefit from antt-PD-1 immune checkpoint blockade and identifying it within IMF confirms those patients as prime candidates for immunotherapy. At the opposite extreme, the IMF classification pinpoints the fibroblast-rich iCMS3_MSS_F tumors, which mirror the prototypical CMS4 mesenchymal subtype with an immunosuppressive, pro-angiogenic microenvironment. These tumors harbor a dense stroma of cancer-associated fibroblasts and myeloid cells, contributing to immune evasion and a propensity for metastasis. Such cases—the poorest-prognosis subset in IMF—may require novel therapeutic approaches in combination with standard therapy to improve outcomes. Importantly, the IMF system also highlights intermediate MSS groups that might otherwise be overlooked; for example, iCMS3_MSS_NF tumors (microsatellite-stable but intrinsic iCMS3 without fibrosis) show transcriptomic and regulatory network similarities to MSI-H tumors. This suggests they could be immunologically primed despite being MSS, raising the prospect of extending immunotherapy or combination strategies to a subset of MSS patients based on IMF features. Overall, by encompassing tumor-intrinsic biology and the tumor microenvironment, the IMF classification provides a more nuanced stratification than iCMS + MSI alone. It defines clinically relevant subgroups with distinct prognoses and likely therapy responses, thereby laying a foundation for more personalized treatment planning in colorectal cancer.

Therapeutically, several approaches have growing evidence. PD-1 monotherapy is the first-line approach for MSI-H/dMMR disease, with superior progression-free survival and durable responses versus chemotherapy and sustained benefit on extended follow-up [14,15,30]. For BRAF-V600E mCRC, encorafenib + cetuximab (with or without a MEK inhibitor) improved outcomes after prior therapy [13,14], and encorafenib + cetuximab + mFOLFOX6 has now demonstrated first-line survival gains, leading to U.S. accelerated approval pending confirmatory evidence [40,41]. Among MSS, RAS-WT, and left-sided tumors, often aligned with canonical epithelial mechanisms, anti-EGFR + doublet chemotherapy confers an OS advantage over bevacizumab-based options, establishing an EGFR-first principle for this clinicogenomic subset [15,42].

By contrast, MSS-iCMS3 remains a central unmet need. The VEGF-TKI/PD-1 doublet regorafenib + nivolumab shows modest activity overall, with responses concentrating in patients without baseline liver metastases, suggesting strong site dependence [33]. Randomized trials to “heat up” unselected MSS CRC, such as the approaches of atezolizumab + cobimetinib and lenvatinib + pembrolizumab, failed to improve survival versus standard options [34,35]. Outside classical ICIs, T-cell-redirecting strategies demonstrate proof-of-mechanism but require toxicity-aware protocols and careful patient selection [36]. Taken together, current data justify biomarker-enriched, site-aware trials for MSS-iCMS3 rather than off-protocol adoption of ICI-based combinations.

The fibrosis (F) axis emerges as both a biological driver of immune exclusion and a therapeutic opportunity. TGF-β-driven CAF mechanisms correlate with desmoplasia and resistance, providing mechanistic rationale for TGF-β-modulating combinations (with PD-1 or cytotoxics) to decompress stroma and enhance T-cell entry [10]. In parallel, FAP-targeted theranostics are progressing from feasibility to structured trials, with early signals of activity and manageable safety in radioligand therapy readouts, while standardization of ligands, dosimetry, and acquisition/interpretation criteria remains a priority before broad clinical use [17]. FAPI-PET complements this agenda by offering a sensitive stromal readout, particularly useful in peritoneal disease, though benign fibro–inflammatory uptake and shorter tumor retention of first-generation tracers require cautious interpretation and, where available, next-generation ligands [16,45,47].

Anatomical organ context measurably shapes immunotherapy readouts and should be prespecified in cohort design and trial eligibility. The liver microenvironment appears specifically permissive to systemic immune suppression, consistent with the distribution of responses in regorafenib + nivolumab studies [33]. Conversely, peritoneal involvement often reflects stroma-rich biology where FAPI-PET is informative for detection and monitoring [16,45]. IMF-aware decision-making should therefore be site-aware, and analyses should report organ-specific efficacy to avoid dilution of context-dependent signals.

Methodologically, reliable single-sample subtyping depends on pre-analytics, proper normalization, and transparent classifier versioning and confidence metrics. It is helpful to co-report the iCMS label with posterior scores or margins, the MSI assay and date, and the F metric and cut-point, together with any discordance across RNA-based and image-based calls [2,18,20]. When bulk and imCMS labels disagree, multiregional sampling with an area-weighted subtype summary offers stronger prognostic performance than raw heterogeneity counts and may be favored in cohort reports and translational studies [21].

Important limitations remain. Cut-point calibration for the F layer, cross-platform comparability, and center-to-center operational variance require cross-laboratory standardization before regulatory-grade deployment. Much of the iCMS/IMF evidence base has been assembled from heterogeneous retrospective cohorts, thus prospective, preregistered evaluations are needed to demonstrate independent clinical utility, especially for MSS-iCMS3 and F-high groups. Finally, pragmatic surrogates such as imCMS and FAPI-PET add operational value but demand site-specific standard operating procedures (SOPs) to mitigate misclassification.

Looking forward, we anticipate biomarker-enriched, site-aware trials for MSS-iCMS3 that integrate (i) boundary-centric spatial biomarkers (e.g., LAMP3^+^ dendritic cell-CXCL13^+^ T-cell niches) as pharmacodynamic readouts, (ii) organ-stratified eligibility (non-liver vs. liver disease), and (iii) mechanism-driven combinations such as angiogenesis modulators, DC/myeloid agents, and T-cell engagers [4,28,33,36]. For F-high IMF groups, priorities include TGF-β-centric combinations with prespecified stromal pharmacodynamics and FAPI-guided theranostics [10,16,17]. Across both, standardized pipelines and version-controlled classifiers should be coupled to preregistered analysis plans, so that the field can close the loop from single-cell insight to clinically actionable stratification at scale.

## 8. Conclusions

CRC requires a stratification system that is biologically faithful yet operational in clinics. Consolidating single-cell and spatial evidence, iCMS offers a principal epithelial axis, and IMF, which layers intrinsic epithelial state with MSI and a quantifiable fibrosis axis, translates that biology into clinically interpretable groups. This review distills those concepts into an analytical/diagnostic pipeline (bulk RNA-based single-sample iCMS calling, routine MSI testing, and fibroblast/fibrosis quantification via EPIC/MCP-counter) with FFPE- and image-based surrogates, multiregional sampling when heterogeneity is suspected, and reporting suggestions so results are reproducible across centers.

Therapeutically, several decisions may be considered for practice: PD-1 blockade as first-line therapy for MSI-H/dMMR disease; encorafenib + cetuximab; and anti-EGFR plus chemotherapy for left-sided, RAS-wild-type tumors. At the same time, MSS-iCMS3 and F-high IMF groups remain an unmet need. Here, the evidence supports biomarker-enriched, site-aware trials.

To accelerate translation, three steps can be recommended: first, adopting a minimum reporting set with version-controlled classifiers, explicit normalization methods, MSI assay details, and a predeclared F-threshold with sensitivity analyses; second, designing prospective, preregistered studies that are organ-stratified, incorporating multiregional sampling when discordance is likely, and paring imaging with tissue to capture stromal dynamics; and third, releasing standardized pipelines and FAIR-compliant data to enable independent verification and, ultimately, companion diagnostic-grade assay development. If these operational commitments are ready, iCMS/IMF can move from a research construct to a central clinical framework that links molecular states, microenvironment, and therapeutic choices—improving decision quality for patients today while creating a durable platform for future biomarker–drug co-development.

## Figures and Tables

**Table 1:** Present evidence summary—recommendations with key evidence and limitations.

Clinical Scenario	Line/Setting	Suggested Regimen	Key Evidence (Trial; Statistics)	Limitations/Notes
MSI-H/dMMR metastatic CRC (mCRC)	First-line	PD-1 monotherapy (pembrolizumab)	KEYNOTE-177: PFS 16.5 vs. 8.2 mo (HR 0.60, 95% CI 0.45–0.79); OS 77.5 vs. 36.7 mo (HR 0.73, 95% CI 0.53–0.99); 5-yr OS 54.8% vs. 44.2%; grade ≥ 3 AEs 22% vs. 67%	OS may be diluted by a 62% crossover (effective crossover); the evidence is mainly based on PD-1 monotherapy
BRAF V600E-mutated mCRC (often pMMR/MSS)	First-line	Encorafenib + Cetuximab + mFOLFOX6	BREAKWATER: PFS 12.8 vs. 7.1 mo (HR 0.53, 95% CI 0.41–0.68; *p* < 0.001); interim OS 30.3 vs. 15.1 mo (HR 0.49, 95% CI 0.38–0.63; *p* < 0.001)	The indication is limited to BRAF V600E; with a 46.1% incidence of serious adverse events, safety management is required. For MSI-H, first-line therapy should still prioritize PD-1.
RAS-WT, left-sided mCRC (MSS/pMMR)	First-line	Anti-EGFR + doublet chemo (e.g., panitumumab + mFOLFOX6)	PARADIGM: Left-sided OS 37.9 vs. 34.3 mo (HR 0.82; *p* = 0.03); ORR 80.2% vs. 68.6%; PFS 13.1 vs. 11.9 mo (HR 1.00). Meta analysis study: Left sided OS HR 0.80 (95% CI 0.71–0.90), PFS NS (HR 0.93)	Left-sided tumors showed concentrated benefit; no OS gain on the right, with PFS often favoring bevacizumab. EGFR-related toxicities (skin rash, hypomagnesemia) require management.
pMMR/MSS—immune checkpoint inhibitor (ICI) combination trial	Post-standard (pretreated)	(mainly negative results)	LEAP-017: lenvatinib + pembrolizumab vs. SOC, OS 9.8 vs. 9.3 mo (HR 0.83) IMblaze370: atezolizumab + cobimetinib vs. regorafenib, OS 8.87 vs. 8.51 mo (HR 1.00, *p* = 0.99)	Unselected pMMR/MSS: no OS benefit in phase III trials; trial enrollment advised considering biomarkers and organ context

Abbreviations: mFOLFOX6, modified fluorouracil/leucovorin/oxaliplatin; SOC, standard of care; ORR, objective response rate; PFS, progression-free survival; mo, month; HR, hazard ratio; CI, confidence interval; OS, overall survival; yr, year; AE, adverse events; NS, not significant.

**Table 2:** Next-generation FAP ligands and FAP-targeted radiotheranostics.

Ligand/Program	Modality and Key Features	Development Status (as of 2025)	Key Data/Notes
FAP-2286	Peptide binder; comparatively longer tumor residence and internalization	Phase I/II ongoing (LuMIERE)	First-in-human studies reported acceptable tolerability and favorable dosimetry; early signals of activity. Ongoing trial evaluates safety, dosimetry, and preliminary efficacy.
OncoFAP-23	Multivalent small molecule designed to improve tumor uptake and retention	Preclinical (clinical entry in preparation)	2024 preclinical work showed higher tumor retention with reduced normal-organ uptake and improved in vivo antitumor effects—addresses short-residence limitation of first-generation tracers.
FAPI tetramers/multimeric optimization series	Multimeric/high-avidity designs to enhance affinity and residence time	Preclinical/translational	Structure-activity optimization reports describe improved kinetics (improved residence and target binding) relative to early monomers; clinical translation pending.
FAPI-46 family	Early-generation small molecules	Case series/small exploratory studies	Compassionate-use and small cohorts suggest feasibility and manageable safety; in some tumors, rapid washout limits delivered dose—motivates next-gen ligands.

## Data Availability

No new data were created or analyzed in this study. Data sharing is not applicable to this article.

## Abbreviations

The following abbreviations are used in this manuscript:

AE	Adverse event
AUC	Area under curve
CAF	Cancer-associated fibroblast
cfDNA	Cell-free DNA
CI	Confidence interval
CIMP	CpG-island methylator phenotype
CMS	Consensus molecular subtypes
CMSFFPE	FFPE-curated CMS classifier
CRC	Colorectal cancer
CRIS	CRC intrinsic subtypes
ctDNA	Circulating tumor DNA
dMMR	Deficient mismatch repair
EC	Encorafenib + cetuximab
ECM	Extracellular matrix
EMT	Epithelial–mesenchymal transition
EPIC	Estimate the proportion of immune and cancer cells
FAPI	FAP-inhibitor
FAPI-PET	FAP-targeted PET
FDA	Food and Drug Administration
FFPE	Formalin-fixed, paraffin-embedded
GR	Growth rate
H&E	Hematoxylin and eosin
HR	Hazard ratio
ICI	Immune checkpoint inhibitor
iCMS	Epithelial–intrinsic consensus molecular subtypes
IHC	Immunohistochemistry
imCMS	Image-based CMS
IMF	Intrinsic subtype-MSI-fibrosis
MCP	Microenvironment cell populations
mCRC	Metastatic CRC
mFOLFOX6	Modified fluorouracil/leucovorin/oxaliplatin
mo	Month
MSI	Microsatellite instability
MSI-H	MSI-high
MSS	Microsatellite-stable
NGS	Next-generation sequencing
NS	Not significant
ORR	Objective response rate
OS	Overall survival
PCR	Polymerase chain reaction
PDO	Patient-derived organoid
PDX	Patient-derived xenograft
PFS	Progression-free survival
pMMR	Proficient mismatch repair
QC	Quality control
RIN	RNA integrity number
SOC	Standard of care
SOP	Standard operating procedure
TMB	Tumor mutational burden
TME	Tumor microenvironment
TRT	Targeted-radiogland therapy
UMI	Unique molecular identifier
WT	Wild-type
yr	Year
